# A Coupled CFD-DEM Study on the Effect of Basset Force Aimed at the Motion of a Single Bubble

**DOI:** 10.3390/ma15155461

**Published:** 2022-08-08

**Authors:** Huiting Chen, Weitian Ding, Han Wei, Henrik Saxén, Yaowei Yu

**Affiliations:** 1 State Key Laboratory of Advanced Special Steel, Shanghai Key Laboratory of Advanced Ferrometallurgy, School of Materials Science and Engineering, Shanghai University, Shanghai 200444, China; 2 Thermal and Flow Engineering Laboratory, Department of Chemical Engineering, Åbo Akademi University, Biskopsgatan 8, FI-20500 Åbo, Finland

**Keywords:** Basset force, CFD-DEM, bubble, unsteady force

## Abstract

The physical meaning of Basset force is first studied via polynomial approximation and the Fourier series representation method. After compiling the Basset force into the coupling interface with Visual C, a dynamic mathematical model is set up to describe the upward motion behavior of a single bubble by adopting the CFD-DEM method. Afterwards, the coupling interface with Basset force proposed in this study is verified experimentally and shows very good agreements. The initial velocity, releasing depth, bubble size, density ratio and viscosity ratio are studied qualitatively due to their great importance to Basset force. The ratio of Basset force to the sum of Basset force and drag force and to buoyancy, ${\vec{F}}_{Ba}$/(${\vec{F}}_{D}$+${\vec{F}}_{Ba}$) and |${\vec{F}}_{Ba}$/${\vec{F}}_{B}$|, are employed to quantify the contribution of Basset force quantitatively. In addition, some instructive outlooks and recommendations on a further development of appropriate and justifiable use of Basset force are highlighted at last.

## 1. Introduction

Multiphase flow plays a critical role in natural and industrial systems, which exists in air bubbles in the ocean [1], sediment transport in rivers [2], the pneumatic transport of solids in the mining industry [3], particle–air systems in the atmosphere [4] and carbon dioxide in methylamine of chemical engineering [5]. Its cross-discipline, richness, diversity and complexity result in the important topics of various branches of science and engineering. Multiphase flow is the commonest cause of a carrier medium (water), where bubbles in water lead to the formation of a discrete phase [6]. Note that the motion of bubbles is very complicated, which is regarded as the deciding factor in controlling mass and heat transfer in multiphase flow. With the development of experimental technology and analytical means, the study on bubble motions tends to focus on the complex flow conditions and accurate description.

In multiphase flow, there are not only constant forces acting on the bubble but also unsteady forces (virtual mass force and Basset force). As one of an unsteady force, the Basset force is owing to the change of relative velocity with time, leading to the lagging development of the boundary layer on the bubble surface. Since its magnitude is directly related to the activity history of the bubble, it is called “History force” [7]. We can trace the research of the Basset force back to the 19th century, and its expression mostly follows the study of Stokes [8] and Basset [9].

(1) $${\vec{F}}_{Ba}=\frac{3}{2}d_{b}^{2}\sqrt{{\pi \rho }_{f}\mu_{f}}\int_{t_{0}}^{t}\frac{\frac{d\left(\vec{u}-\vec{v}\right)}{dt^{\prime }}}{\sqrt{t-t^{\prime }}}dt^{\prime }$$

where the subscripts *b* and *f* mean the bubble and fluid phase, $d_{b}$ represents the bubble/particle diameter, *t* is time, $t_{0}$ is the initial time, $\vec{u}$ and $\vec{v}$ denote the fluid and bubble/particle velocity, respectively, and $\rho_{f}$ and $\mu_{f}$ represent the fluid density and dynamic viscosity, respectively. Odar and Hamilton [10] later extended the Basset–Boussinesq–Oseen equation (BBO equation) to the influence of a positive acceleration term for the linear and unrestricted motion of the sphere. The correction coefficient, $C_{B}$, was introduced by Odar and Hamilton [10]; hence, one can write

(2) $${\vec{F}}_{Ba}=\frac{3}{2}d_{b}^{2}\sqrt{{\pi \rho }_{f}\mu_{f}}C_{B}\int_{t_{0}}^{t}\frac{\frac{d\left(\vec{u}-\vec{v}\right)}{dt^{\prime }}}{\sqrt{t-t^{\prime }}}dt^{\prime }$$

where $C_{B}=0.48+\frac{0.52A_{n}{}^{3}}{{\left(1+A_{n}\right)}^{3}}$ and $A_{n}=\frac{\left|\frac{d\left(\vec{u}-\vec{v}\right)}{dt}\right|}{{\left(\vec{u}-\vec{v}\right)}^{2}}d_{b}$. Therefore, the physical meaning of Basset force is well understood: Basset force represents the time integral of the additional viscous force on the particle during the unsteady motion, which describes the diffusions of vorticity around the particle during its whole history [11].

A mathematical analysis of the expression for the further study is necessary as provided in the following. Relative acceleration, $\frac{d\left(\vec{u}-\vec{v}\right)}{dt^{\prime }}$, is defined as g($t^{\prime }$) and *B* denotes $\frac{3}{2}d_{b}^{2}\sqrt{{\pi \rho }_{f}\mu_{f}}$. Therefore, the expression of Basset force is evaluated in Equation (3).

(3) $${\vec{F}}_{Ba}=BC_{B}\int_{t_{0}}^{t}\frac{g(t^{\prime })}{\sqrt{t-t^{\prime }}}dt^{\prime }$$

In order to describe two states of motion, two approaches are employed to the expression now: (a) polynomial approximation and (b) Fourier series representation.

(a) In the method of polynomial approximation, the particle moves in a straight line with acceleration relative to the fluid: g($t^{\prime }$) = b${t^{\prime }}^{n}$, b ∈ C, n ∈ N*. The expression of Basset force is evaluated as follows.

(4) $${\vec{F}}_{Ba}=BC_{B}\int_{t_{0}}^{t}\frac{b{t^{\prime }}^{n}}{\sqrt{t-t^{\prime }}}dt^{\prime }$$

Different values of n lead to various conditions, as shown in Table 1.

(b) In the method of Fourier series representation, the particle moves in a linear vibration: Fourier series is applied in g($t^{\prime }$).

(5) $$g(t^{\prime })=A_{n}e^{jnt^{\prime }}$$

where $A_{n}$ expresses the amplitude, and *n* denotes the number of waves; hence, one can write

(6) $${\vec{F}}_{Ba}=BA_{n}C_{B}e^{jnt}\int_{t_{0}}^{t}\frac{e^{-jn\left(t-t^{\prime }\right)}}{\sqrt{t-t^{\prime }}}dt^{\prime }$$

Set θ equal to $\sqrt{n\left(t-t^{\prime }\right)}$, and the expression can be evaluated as ${\vec{F}}_{Ba}=\frac{2BA_{n}C_{B}e^{jnt}}{\sqrt{n}}\int_{t_{0}}^{\sqrt{nt}}e^{-j\theta^{2}}d\theta$. As $\sqrt{nt}$ approaches infinity and $\int_{0}^{\infty }e^{-j\theta^{2}}d\theta$ equals (1−j)$\sqrt{\frac{\pi }{8}}$, the final form is ${\vec{F}}_{Ba}=BA_{n}C_{B}e^{j\left(nt-\frac{\pi }{4}\right)}\sqrt{\frac{\pi }{n}}$. If *n* is equal to a constant and *t* is large enough (phase lags $\frac{\pi }{4}$), the amplitude will grow $BC_{B}\sqrt{\frac{\pi }{n}}$ times $A_{n}$. If *n* tends to infinity, ${\vec{F}}_{Ba}$ will equal zero, where the Basset force driven by the high-frequency pulsation of flow is very small and plays an important role in acoustics. On this point, it is quite consistent with the conclusion from Dodemand et al. [12]. To this end, the physical meaning and mathematical analysis of Basset force are presented clearly.

The BBO equation proposed independently by Basset [7], Boussinesq [13] and Oseen [14] can describe the unsteady motion of a single particle at a low Reynolds number. For the motion of spherical particles in a uniform flow, the BBO equation is given as

(7) $$\frac{\pi }{6}\rho_{b}d_{b}^{3}\frac{d\vec{v}}{dt}=C_{D}\frac{1}{8}\rho_{f}\pi d_{b}^{2}\left(\vec{u}-\vec{v}\right)\left|\vec{u}-\vec{v}\right|-\frac{\pi }{6}d_{b}^{3}\nabla p+\frac{\pi }{12}\rho_{f}d_{b}^{3}\frac{d}{dt}\left(\vec{u}-\vec{v}\right)+\frac{3}{2}d_{b}^{2}\sqrt{{\pi \rho }_{f}\mu_{f}}\int_{t_{0}}^{t}\frac{\frac{d\left(\vec{u}-\vec{v}\right)}{dt^{\prime }}}{\sqrt{t-t^{\prime }}}dt^{\prime }+\underset{k}{\sum }{\vec{F}}_{k}$$

where $\rho_{b}$ represents the bubble/particle density, $C_{D}$ means the drag coefficient, and ∇p denotes the pressure gradient. The left-hand side of Equation (7) expresses the particle momentum change rate, while the right-hand side includes the Stokes viscous drag, pressure gradient force, virtual mass force, Basset force and other forces (such as gravity), respectively.

With the increasingly accurate description of bubble/particle motion characteristics under complex flow conditions, the Basset force draw much attention. For bubbles/particles moving at variable speed in non-uniform fluid, the Basset force represents the influence of unsteady viscosity in the process of motion, which should not be ignored. Nonetheless, additional difficulties arise due to the complicated origin and calculation of Basset force. As seen in Equation (7), the BBO equation becomes an integro-differential equation because of the presence of the Basset force term, leading to a much more demanding solution than that of an ordinary differential equation. Therefore, it is a common practice to inappropriately use and even ignore the Basset force term in the literature, but it is not at all justifiable [15,16]. Some scholars suggested that it can be ignored, while others argued that it cannot be. Using the method of direct numerical simulation (DNS), Elgobashi and Truesdell [17] studied the dispersion of solid particles in decaying isotropic turbulence and reported that the Basset force is negligible for any cases. They reported that the drag and buoyancy forces are dominant in the gravity direction, and the drag and Basset forces are the main forces in the lateral directions. Choosing a method of linearization with introducing a complex wave-number, Dodemand et al. [12] studied the motion characteristics of particles in oscillating fluid and argued that the Basset force can be ignored when the oscillation angular frequency is extremely small or large for the periodic oscillatory flow. They argued that the unsteady terms are of great importance when the density ratio (mass density of the particle divided by the mass density of the fluid) becomes small. Armenio and Fiorotto [18] carried out DNS for a wide range of density ratios and observed that the Basset force should be considered in any circumstances with respect to other forces. Combining the third-order quadrature scheme with a third order Adams–Bashforth–Moulton predictor–corrector scheme, Daitche and Tél [11] investigated the effect of Basset force on particle advection and suggested that the particle clustering and the caustic formation are greatly reduced. Olivieri et al. [19] addressed the effect of the Basset history force on the dynamics of small particles transported in homogeneous and isotropic turbulence and delineated that the Basset force term reduces the small-scale clustering typical of inertial particles and is particularly relevant during rare strong events. As mentioned above, all the studies on the Basset force focused on the solid particles, and the bubbles with small mass still lack study. However, the unsteady terms of bubbles with a small density ratio to the fluid is of great importance. Moreover, only the density ratio is discussed in the previous studies, and a complete solution involving all potential influence factors is not yet available, such as the initial velocity of the bubble, the bubble-releasing depth, bubble size, and the viscosity ratio of the two phases. What is more, the use of the Basset force should be considered under the practical condition.

In practical engineering, bubble/particle relative velocity and its derivative to time are the quantities to be solved. The integrand function in the expression of Basset force is unknown, so it is impossible to solve it directly. Additionally, the integrand function has a singularity on the integral interval, and it belongs to a generalized integral. Hence, numerical study is necessary. In order to investigate the bubble motion more accurately, some numerical simulation methods have been employed, and two main approaches are Eulerian–Eulerian and Eulerian–Lagrangian. Although Eulerian–Eulerian approaches have lower computational complexity, its simulation results do not show the detailed information as Eulerian–Lagrangian approaches do. To our knowledge, as the Eulerian–Lagrangian method, the “computational fluid dynamics-discrete element method” (CFD-DEM) can directly trace the particles, droplets and bubbles, which is widely employed to simulate the multiphase flow, including the residence time distribution of particles in the reactor [20], mixing and segregation of the multi-particle size system [21], electrostatic force between particles [22], and wear of the particles and reactor [23]. In the research method aspect, most studies have been carried out via DNS and theoretical analysis, and there is not yet a coupled CFD-DEM study on the effect of Basset force aimed at a single bubble. Therefore, in this paper, attention is given to when and where the Basset force should be ignored via a coupled CFD-DEM approach. It is noteworthy that our object in this research is a single bubble, and we will study the bubble swarms or a group of bubbles rising through the systems in our future studies. This study should be regarded as a first step toward the justifiable and appropriate use of Basset force. Because the qualitative and quantitative conclusions above do provide a reference for related theories and applied research under certain conditions, this can be a framework for us to verify the force contribution.

In this work, employing the CFD-DEM method by Fluent software and EDEM, this paper constructs a dynamic coupling mathematical model to describe the upward movement of the bubble considering the effect of Basset force in Section 2. Subsequently, the accuracy of the model with Basset force is presented in Section 3 and verified experimentally. In order to provide a reference for related theories and applied research under certain conditions, the effect of Basset force on a single bubble motion is studied qualitatively and quantitatively in Section 4. Finally, conclusions and recommendations on Basset force for a further study are drawn in Section 5.

## 2. Methodology

As for the motion of a single bubble in a multiphase system, it is essential for us to take full consideration on the coupling relationship in order to realize the unsteady coupling process [24]. Therefore, in this work, a coupled model is constructed with Fluent software (ANSYS Inc., Canonsburg, PA, USA) for the continuous phase and EDEM software (DEM Solutions Ltd., Edinburgh, UK) for the discrete phase, where the fluid and the bubble are discretized in the Eulerian and Lagrangian framework, respectively.

**Figure 1:**
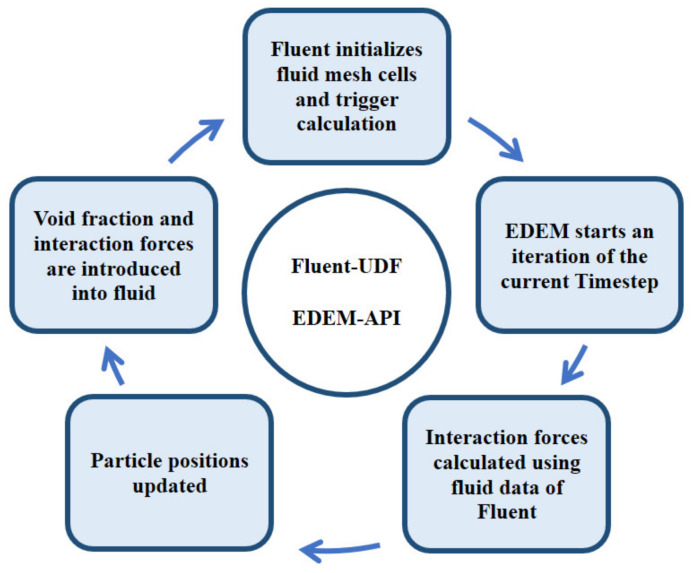
Fluent–EDEM coupling scheme.

The reason why we chose EDEM software to study the bubble motion is that it can evaluate the interaction forces between bubbles and consider the coalescence and breakup accurately in a DEM way [25,26]. More importantly, a future aim of this study is to expand the objects to focus on the bubble swarms or a group of bubbles rising through the systems in our later research. Therefore, EDEM software using the DEM method is feasible, which has been employed to study the bubble motion in previous studies [25,26,27]. As seen in Figure 1, Fluent–EDEM coupling is a transient bi-directional data transmission process, which can be realized by a coupling interface written with Visual C. This method can simulate particle motion and interaction with the flow field more accurately. Firstly, flow information of a time step is calculated by Fluent, and EDEM starts an iteration in the meantime. The bubble position, motion, volume and temperature are transferred by the coupling interface to Fluent to calculate the interaction between the bubble and fluid. The effect of fluids on the bubble is transmitted by the coupling interface, while the effect on fluids acts on itself through the momentum source term. This can ensure the transient simulation of the whole process with the iterative step-by-step approach.

### 2.1. Discrete Phase

The translation and rotation of the bubble are solved by Newtonian motion equations in EDEM, and the governing equations are as follows.

(8) $$m_{b}\frac{d\vec{v_{i}}}{dt}=\sum_{j}{\vec{F}}_{c,ij}+\sum_{k}{\vec{F}}_{nc,ik}+{\vec{F}}_{bf}+{\vec{F}}_{G}$$

(9) $$I_{b}\frac{d{\vec{\omega }}_{i}}{dt}=\sum \left({\vec{M}}_{r,ij}+{\vec{M}}_{t,ij}\right)$$

where $m_{b}$, $I_{b}$, $\vec{v_{i}}$ and ${\vec{\omega }}_{i}$ denote the mass, the moment of inertia, the translation velocity and the angular velocity of the bubble, respectively. In the right side of Equation (8), ${\vec{F}}_{c,ij}$, ${\vec{F}}_{nc,ik}$, ${\vec{F}}_{bf}$ and ${\vec{F}}_{G}$ represent the contact force, the noncontact force, the bubble–fluid interaction force and the gravity force. In addition, ${\vec{M}}_{r,ij}$ and ${\vec{M}}_{t,ij}$ express the rolling and tangential friction moments. Finally, it is appropriate to point out here that the normal force and tangential force are solved by the Tsuji model [28] and Mindlin model [29,30].

### 2.2. Continuous Phase

The Eulerian multiphase model in Fluent is chosen, which is mass conservative and allows for the modeling of multiple separate yet interacting phases [31]. In this paper, a Eulerian treatment is used for the primary phase (fluid) and discrete phase (the bubble), and the volume fraction of phase-q is defined as $\alpha_{q}$. The volume of each phase-q ($V_{q}$) is defined by

(10) $$V_{q}=\int_{V}^{}\alpha_{q}dV$$

where the volume fraction of all phases adds up to 1; that is, $\alpha_{b}=1-\alpha_{f}$. The effective density is $\rho =\alpha_{f}\rho_{f}+\alpha_{b}\rho_{b}$ and the effective viscosity is μ = ${\;\alpha }_{f}\mu_{f}+\alpha_{b}\mu_{b}$.

The mass and momentum conservation equations of phase-q are [32]

(11) $$\frac{\partial \alpha_{q}}{\partial t}+\nabla \cdot \left(\alpha_{q}{\vec{u}}_{q}\right)=0$$

(12) $$\rho_{q}\left(\frac{\partial }{\partial t}\left(\alpha_{q}{\vec{u}}_{q}\right)+\nabla \cdot \left(\alpha_{q}{\vec{u}}_{q}{\vec{u}}_{q}\right)\right)=-\alpha_{q}\nabla p+\nabla \cdot {\vec{\tau }}_{q}+\alpha_{q}\rho_{q}\vec{g}+{\vec{f}}_{\mathrm{bf}}$$

where p is the pressure shared by all phases, and the stress–strain tensor of phase-q (${\vec{\tau }}_{q}$) is defined as

(13) $${\vec{\tau }}_{q}=\alpha_{q}\mu_{q}\left(\nabla {\vec{u}}_{q}+{\left(\nabla {\vec{u}}_{q}\right)}^{T}\right)+\alpha_{q}\left(\lambda_{q}-\frac{2}{3}\mu_{q}\right)\nabla \cdot {\vec{u}}_{q}\overset{=}{I}$$

where $\mu_{q}$ and $\lambda_{q}$ denote the shear and bulk viscosity of phase-q, respectively, $\overset{=}{I}$ is the identity matrix, and ${\vec{f}}_{\mathrm{bf}}$ expresses the reacting force of bubble–fluid interactions as provided in the following Section 2.3.

### 2.3. Bubble–Fluids Interactions

In the CFD-DEM model, the expression of each interaction force contained in the term of bubble–fluids interaction needs to be given. The interaction forces include drag, pressure gradient force, viscous stress force, Reynolds stress force, capillary force, virtual mass force, Saffman lift force, Magnus force and Basset force. These forces are compiled into the coupling interface with Visual C, whose models are precisely provided in the Supplementary Materials. It is appropriate to emphasize that the Basset force is commonly overlooked in traditional calculation; hence, the accuracy of new coupling interface considering the Basset force should be verified in the following Section 3 first.

## 3. Validation Tests

In this section, the data of flow behavior in a gas–liquid system are gained experimentally, and the CFD-DEM model in Section 2 is solved by coupling Fluent and EDEM software with the coupling interface after compiling Basset force code into the coupling interface. Finally, the experimental and simulation results are compared to verify the accuracy of the coupling model.

### 3.1. Experimental

**Figure 2:**
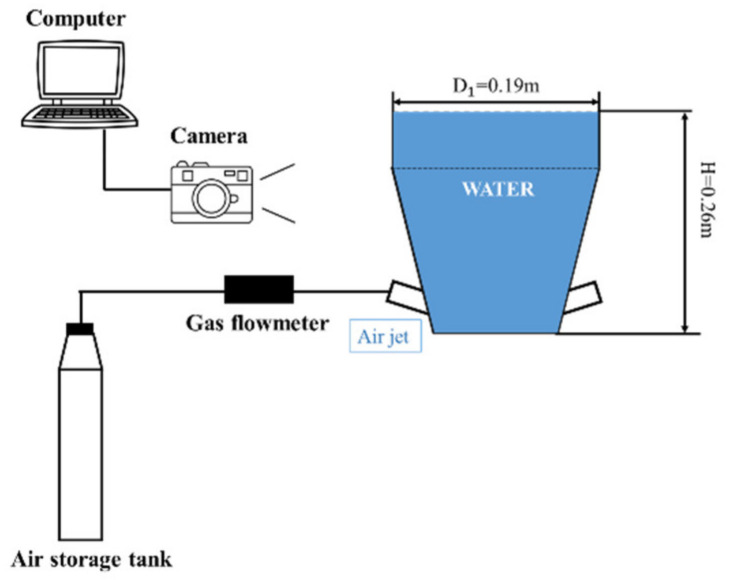
Schematic of experimental setup.

The schematic of the experimental apparatus is shown in Figure 2. The flow was processed by the bottom-blown device made of organic glass; hence, the flow behavior in the gas–liquid system was recorded with the help of photographing equipment clearly. The apparatus filled with quiescent water was composed of a straight section and conical section, and its basic structure diagram is shown below. The straight section is connected with the conical section by flanges. The inner diameter of the straight section was 0.19 m and the liquid level was 0.26 m high. Four symmetry air jets were set in the conical section, considering the bubble swarms or multiple groups of bubbles rising through the systems in our future studies. The values of medium density and viscosity are provided in Table 2. Experiments were carried out under five different flow rates: 132 L/h, 176 L/h, 220 L/h, 264 L/h and 308 L/h. For more experimental details, see previous research [33].

### 3.2. Simulation Conditions

Four bubble/particle generators (as four air jets) are symmetrically set 0.07 m above the bottom of the domain in EDEM. The gravitational field points in the negative y-direction. Water and the bubble are considered as the primary and secondary phase, respectively. The parameters of physical properties used are the same as those in Table 2, and the bubble diameter is 1 mm.

For boundary conditions, all walls adopt a no-slip wall condition, and four bubble/particle generators are set for the inlet condition in EDEM, while the pressure outlet and an atmosphere boundary condition are set for outlet in Fluent. The bubble boundary condition for the outlet boundary is set as escape type, while the other wall boundary is set as the reflective type.

The bubble Reynolds number is less than 500 and the initial velocity of the liquid phase induced by the single rising bubble is close to zero; hence, it is assumed for the single bubble flow to be laminar.

The scheme of pressure–velocity coupling is phase-coupled SIMPLE, and a second-order upwind scheme is chosen for solving the momentum and volume fraction equations. The gradient is evaluated by least squares cell based. The simulations were carried out using ANSYS Fluent 19.0 and EDEM 2019. The fluid time step should meet the Courant–Friedrichs–Lewy condition ($\Delta t<\frac{\Delta x}{v_{max}}$), where Δt represents time step, Δx means the size of the computational grid, and $v_{max}$ denotes the maximum fluid velocity in the computational domain [34]. The criterion to set up a time step in a DEM simulation is that it has to be less than the time it would take for the Rayleigh wave to travel the minimum size particle in the assembly. This critical time step is called a Rayleigh time step, and using this method is a generally accepted way of approximating a suitable time step which gives a stable numerical solution in DEM [35,36]. Considering the extremely low mass of the single bubble, the momentum transfer has a great impact on the bubble velocity, it is suggested that the setting of the Fluent time step is the same as that of EDEM. In this work, the time step in both Fluent and EDEM is set to 4.0 × 10^−7^ s.

It is necessary to make the following assumptions in order to simplify the model: (a) in order to exclude the effect of bubble shape, the bubble is fixed to be 1 mm in this work, considering that a 1 mm bubble with a Reynolds number smaller than 300 and Tadaki number smaller than 1 remains to be spherical [37]. (b) It has been identified that interfacial area transport equations (IATE) can handle all bubbles in two groups: the spherical/distorted bubble group and the cap/slug bubble group [38,39,40]. Moreover, 1 mm bubbles belong to Group I bubbles (spherical/distorted bubbles) in bubbly flow, which are treated in one-group IATE rather than two-group IATE [24,41]. Therefore, the coalescence and breakup of 1 mm bubbles are neglected in the dilute phase flow, and it is reasonable [24]. (c) Water is turbulent incompressible Newtonian fluid and initially at rest.

**Figure 3:**
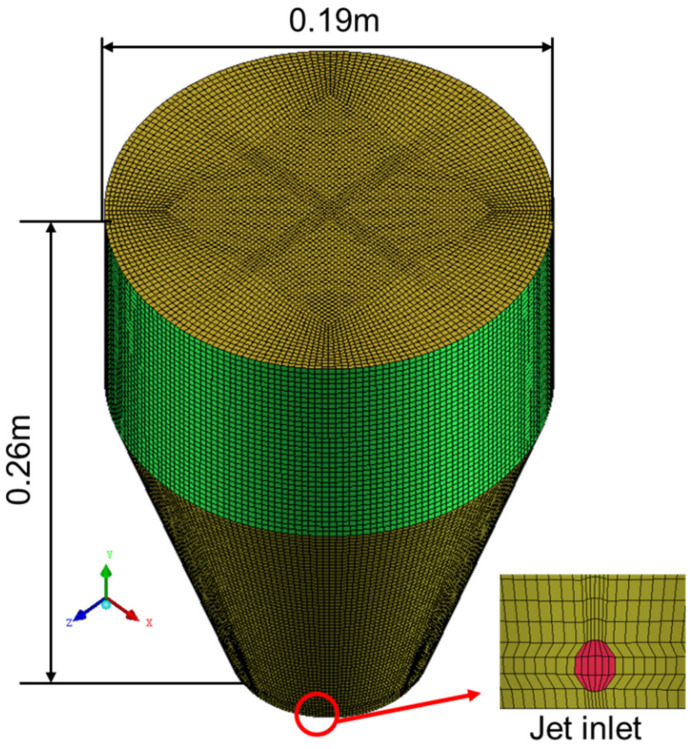
Structured meshes of the computational domain.

It has been identified that when the computational grid size is too small, the uncharacteristic behavior of the bubbly flow is obtained and the numerical instability is occurred [42,43]. Therefore, in order to calculate the bubble–fluids interaction forces exactly, grid sizes are required to be larger than the size of the discrete phase. We performed an O-type structured grid and densification at the bubble injection and trajectory, as seen in Figure 3. A test of mesh independence is carried out, and the information of meshes is provided in Table 3. In the fourth column, the Grid Convergence Index (GCI) proposed by Roache to assess the effect of the grid resolution quantitatively is provided, which represents the result of the mesh tests [44,45]. The GCI value reduces with grid refinements. The GCI value of meshes 1–3 is relatively small (0.52–2.81%); this suggests that the discretization error is relatively small.

**Figure 4:**
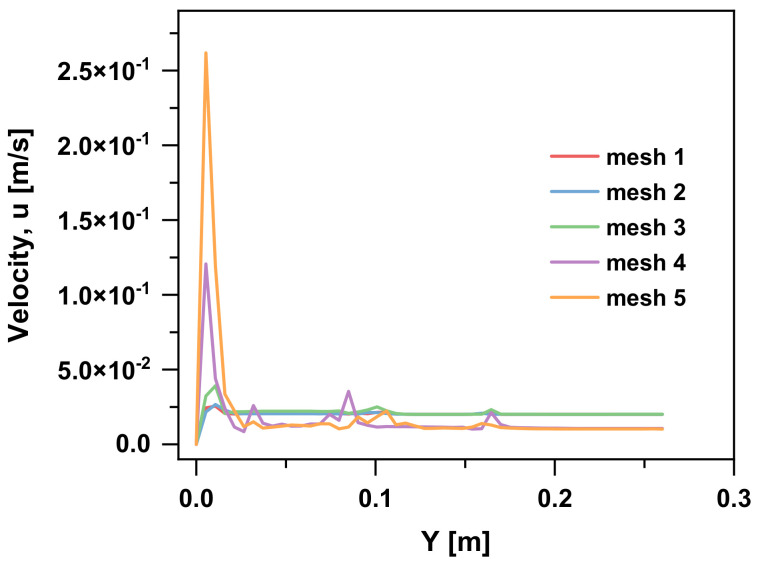
Velocity of the flow along y-axis of bubble rising trajectory at 5 s.

After the independence test, we found that there was no difference in the bubble velocity among the meshes; hence, we focus on the velocity field. The velocity of the flow along the y-axis of the bubble-rising trajectory at 5 s is shown in Figure 4. Meshes 1–3 can accurately predict the flow velocity, whose deviation percentage is small, and the velocities are almost of the same order of magnitude. Compared with fine meshes, mesh 4 and 5 differ greatly in velocity, where the velocities calculated by them are one-magnitude-order larger than that of meshes 1–3. Generally, the grid size must be small enough to ensure numerical stability and accuracy in the transmission of interaction forces, but it should also be sufficiently large to ensure a reasonable analysis time. (Because the smaller the grid size, the longer the simulation time as the computer has to do more calculations.) Based on the above discussion, we believe that meshes 4 and 5 are not desirable due to the inaccurate results induced by the extremely large grid size. Therefore, mesh 3 with a 1.5 mm grid size is selected for later calculation, which can not only better describe the particle and flow field velocity but also has no excessive amount of calculation.

### 3.3. Results

**Figure 5:**
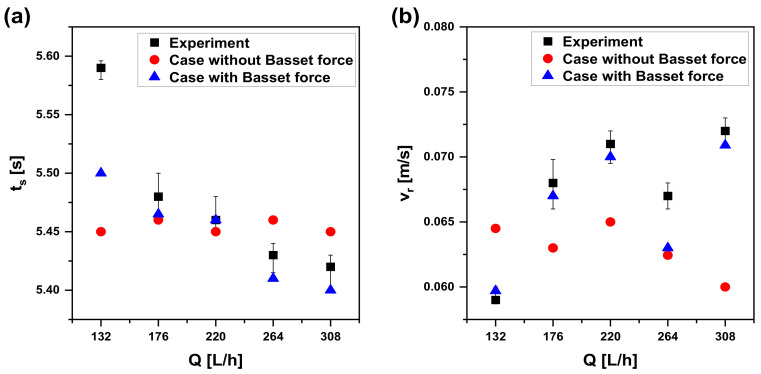
Comparison between the cases and experiment: (**a**) time to reach liquid surface under different flow rates; (**b**) rising velocity under different flow rates.

**Figure 6:**
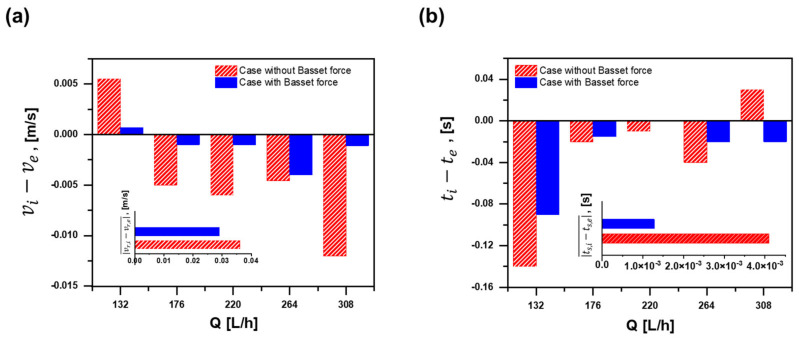
Error with the experiment: (**a**) time to reach liquid surface; (**b**) rising velocity.

The first-generated bubble is chosen for the comparison at first, and a group of bubbles rising through the systems are verified later. As shown in Figure 5a, both of the cases with Basset force and the experiment have an ascent after an initial decline trend in time to reach the liquid surface ($t_{s}$). As for the rising velocity ($v_{r}$) in Figure 5b, both of them increase and then decline at a low velocity level. Therefore, trends of $t_{s}$ and $v_{r}$ are in better agreement with the experimental data after considering the effect of Basset force. Instead, without Basset force, $t_{s}$ and $v_{r}$ are relatively insensitive to different flow rates (Q), and it cannot indicate the practical motion of a bubble under different Q values. The error with the experiment is provided in Figure 6 by a further study. $t_{i}-t_{e}$ and $v_{i}-v_{e}$ represent the differences between the simulation and the experiment, and their absolute average value ($\bar{\left|t_{s,i}-t_{s,e}\right|}$ and $\bar{\left|v_{r,i}-v_{r,e}\right|}$) is illustrated in the inset. Obviously, the simulation data with Basset force have less error than the one without it. Likewise, other bubbles are compared in the same way, and all the bubbles with Basset force show very good agreements with the experimental ones. Therefore, the CFD-DEM model considering Basset force in this paper is reliable for later study aimed at a single bubble.

## 4. Discussion

Based on the analysis of its expression in Section 1, we will focus on some potential influence factors, such as the initial velocity of the bubble, bubble-releasing depth, bubble size, density ratio and viscosity ratio of two phases. In the follow-on discussions, the effect of Basset force on the motion of a single bubble is studied qualitatively and quantitatively by performing the simulations with and without the Basset force code for each parameter set, and only one bubble is released in every case. That is, current studies are beginning to place a greater emphasis on the conditions under whether the effects of Basset force should be ignored or not in the calculation of multiphase flow. Moreover, the effect of Basset force on the bubble motion can be evaluated by the absolute difference in rising velocity between the case with and without Basset force.

For the later study on the long-time calculation of Basset force in this section, an extended model with a height of 9 m is established based on the previous computational domain with the height of 0.26 m, and this model passes the validation test. In this section, the ratio of Basset force to the sum of Basset force and drag force and to buoyancy, ${\vec{F}}_{Ba}$/(${\vec{F}}_{D}$+${\vec{F}}_{Ba}$) and |${\vec{F}}_{Ba}$/${\vec{F}}_{B}$|, are employed to quantify the contribution of Basset force quantitatively.

### 4.1. Initial Velocity Effects

**Figure 7:**
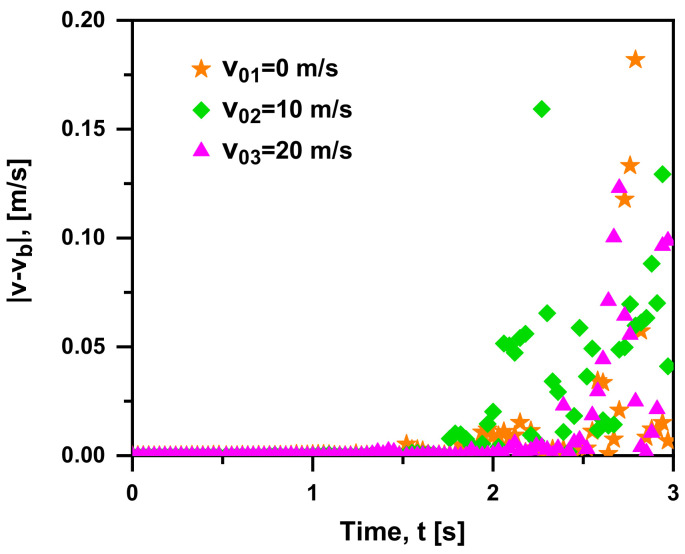
Absolute difference in rising velocity between the case with and without Basset force at three initial velocities ($d_{b}$ = 1 mm, $\rho_{f}$ = 1000 kg/m^3^, $\rho_{b}$ = 1.29 kg/m^3^ in quiescent water).

As seen in Figure 7, the higher the initial velocity ($v_{0}$) is, the smaller |$v-v_{b}$| is. When the initial velocity equals zero, the relative velocity and acceleration are zero at the initial time, so does the Basset force. It corresponds to the special case (b = 0 in ${\vec{F}}_{Ba}=2\mathrm{bB}C_{B}t^{1/2}$), as discussed in Table 1. As the buoyancy accelerates the bubble, the slip velocity is generated and Basset force is regarded as a resistance. In the case of $v_{0}$ = 10 m/s or even 20 m/s, the acceleration is positive and the Basset force is less than zero (as a resistance), delaying the bubble acceleration process. In other words, the Basset force is a matter of less significance to the bubble with a higher initial velocity. This is because a greater initial velocity may lead to a larger slip velocity and momentum, and there are slip velocity terms ($\vec{u}-\vec{v}$) in both Basset force and drag force formulas. Known as a larger order of magnitude than Basset force, drag force is a more dominant force in the motion of the bubble with higher initial velocity. Furthermore, |$v-v_{b}$| increases significantly with time accumulation, and so does the effects of Basset force on the bubble motion as well. As seen from its expression, Basset force is the time integral of the additional viscous force; hence, we are expected to be interested in the bubble’s long-time motion considering Basset force. The bubble accelerates and the change of Basset force directly affects the bubble’s acceleration in the early stage. During the rising process, there is little difference in velocity and the instantaneous velocity fluctuates periodically up and down. Therefore, Basset force is of great importance in the acceleration stage. The influence on bubble motion increases with time and it cannot be overlooked until reaching stability, where its state corresponds to ${\vec{F}}_{Ba}=\frac{2^{k+1}\cdot k!}{1\cdot 3\cdot 5\cdot \cdot \cdot \left(2k+1\right)}bBC_{B}t^{\left(k+1\right)/2}$, as analyzed in Table 1. When buoyancy, drag force, gravity, Basset force and other forces reach a balanced point, the bubble moves at a uniform speed (so-called “suspension velocity”) until the liquid level. This equilibrium state corresponds to the case of n = 0 in Table 1. That is, the expression of Basset force is evaluated into ${\vec{F}}_{Ba}=2\mathrm{bB}C_{B}t^{1/2}$ by polynomial approximation, and the case of b = 0 represents the stable uniform motion of the bubble. In this event, the bubble moves at a uniform speed relative to the fluid and Basset force equal to zero. Finally, it is concluded that the rising consists of three stages: velocity increasing zone, velocity decreasing zone and steady rising zone.

**Figure 8:**
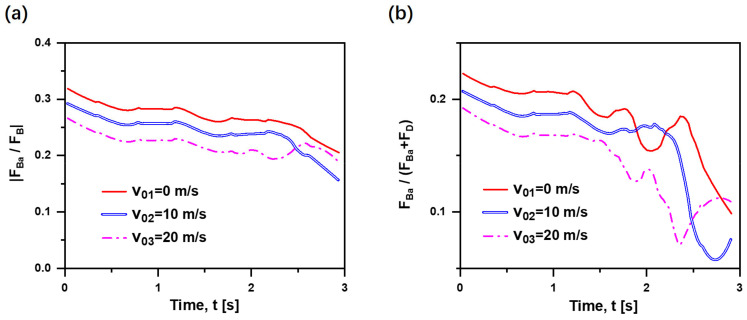
(**a**) $\left|{\vec{F}}_{Ba}/{\vec{F}}_{B}\right|$; (**b**) ${\vec{F}}_{Ba}/\left({\vec{F}}_{D}+{\vec{F}}_{Ba}\right)$ over time at three initial velocities ($d_{b}$ = 1 mm, $\rho_{f}$ = 1000 kg/m^3^, $\rho_{b}$ = 1.29 kg/m^3^ in quiescent water).

On the whole, |${\vec{F}}_{Ba}$/${\vec{F}}_{B}$| within a narrow range (15–30%) decreases with time, as seen in Figure 8a. That is, Basset force has less influence on the bubble motion with time accumulation, and |${\vec{F}}_{Ba}$/${\vec{F}}_{B}$| shows a decreasing trend as the initial velocity increases. As shown in Figure 8b, viscous force and Basset force are two main resistances in the rising process, which are comparable in the same magnitude as a whole. Therefore, the contribution of Basset force on the resistance, ${\vec{F}}_{Ba}$/(${\vec{F}}_{D}$+${\vec{F}}_{Ba}$), can be obtained. ${\vec{F}}_{Ba}$/(${\vec{F}}_{D}$+${\vec{F}}_{Ba}$) decreases with time, and its range varies greatly within 1–30%. As the initial velocity increases, the value of ${\vec{F}}_{Ba}$/(${\vec{F}}_{D}$+${\vec{F}}_{Ba}$) tends to go smaller. This is due to the stable state of the bubble in the late rising process, where the Basset force becomes less dominant. In particular, the lines cross in Figure 8 is because the forces on the bubble gradually approach a balanced point in the late stage of bubble rise (t > 2 s), the Basset force is evaluated into $F_{Ba}=2\mathrm{bB}C_{B}t^{1/2}$ by polynomial approximation, and the case of b = 0 represents the stable uniform motion of the bubble. That is, the bubble moves at a uniform speed relative to the fluid and the Basset force is equal to zero in this event. Obviously, in the case of t > 2 s and $v_{0}$ ≥ 20 m/s, the contribution of Basset force to the total resistance (i.e., the value of ${\vec{F}}_{Ba}$/(${\vec{F}}_{D}$+${\vec{F}}_{Ba}$)) is responsible for less than 10% approximately, where the effect of Basset force can be ignored due to its smaller order of magnitude.

### 4.2. Releasing Depth Effects

**Figure 9:**
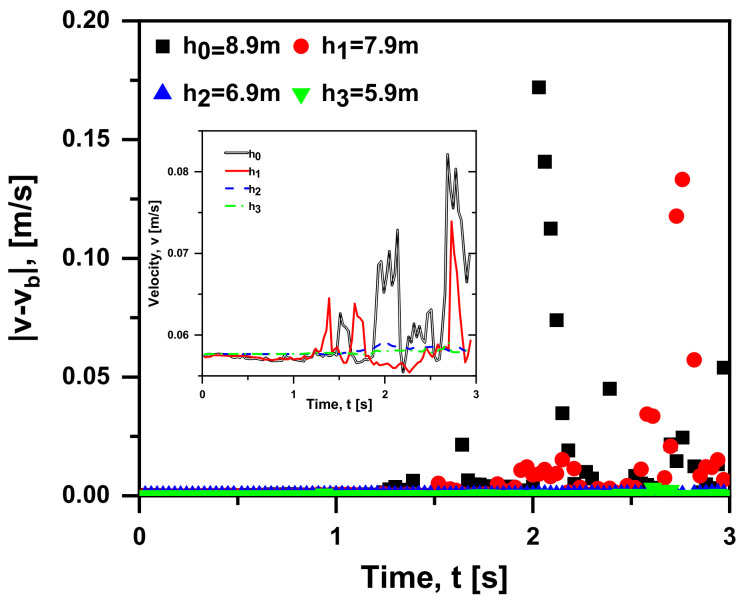
Absolute difference in rising velocity between the case with and without Basset force at four releasing depths; inset: bubble velocity of the case with Basset force ($v_{0}$ = 0 m/s, $d_{b}$ = 1 mm, $\rho_{f}$ = 1000 kg/m^3^, $\rho_{b}$ = 1.29 kg/m^3^ in quiescent water).

As seen in Figure 9, the bubble is released 8.9 m, 7.9 m, 6.9 m and 5.9 m away from the liquid level, respectively. It is clearly evident that the shallower the releasing depth (h) is, the smaller the absolute difference in rising velocity between the case with and without Basset force (|$v-v_{b}$|). This is because the shallower releasing depth causes the smaller pressure at the initial position and pressure gradient force, leading to a smaller initial acceleration and less impact of Basset force on the bubble motion. Significantly, when the bubble is released with the distance less than 6.9 m from the liquid level, |$v-v_{b}$| tends to be zero entirely. Therefore, the effect of Basset force can be ignored in the case of h < 6.9 m. Furthermore, as seen in the inset, the rising velocity becomes more gentle with the decreasing of releasing depth. The lower fluctuation of the case with shallower releasing depth is due to the smaller pressure gradient force, leading to a smaller initial acceleration. Hence, it is easier for the bubble to reach the balance point in this instance.

### 4.3. Bubble Size Effects

**Figure 10:**
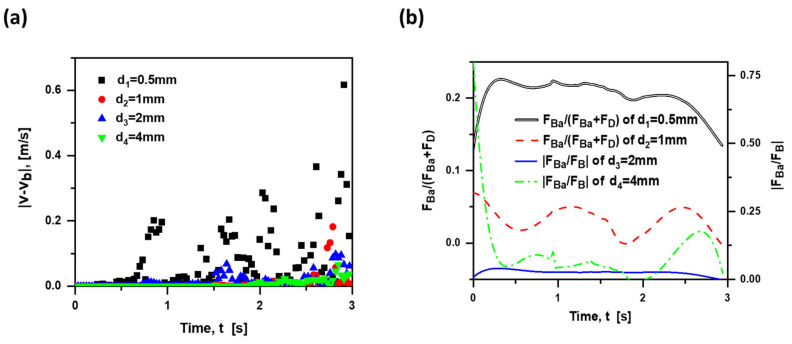
(**a**): Absolute difference in rising velocity between the case with and without Basset force of four diameters; (**b**): ${\vec{F}}_{Ba}$/(${\vec{F}}_{D}$ +${\vec{F}}_{Ba}$ ) and |${\vec{F}}_{Ba}$ /${\vec{F}}_{B}$ | over time at four diameters. ($v_{0}$ = 0 m/s, $\rho_{f}$ = 1000 kg/m^3^, $\rho_{b}$ = 1.29 kg/m^3^ in quiescent water).

Armenio and Fiorotto [18] reported that the importance of Basset force is dependent on particle size. In this paper, four diameters are considered: 0.5 mm, 1 mm, 2 mm and 4 mm. As shown in Figure 10a, the velocity gap decreases with the diameter increasing, and so does the effect of Basset force on the bubble motion. Note that not all Basset forces in these cases are regarded as the resistance. For the case of $d_{4}$ = 4 mm in the inset, the bubble with a relatively large size may have a large buoyancy, leading to a great acceleration and negative Basset force at the beginning. In this instance, it is faster for the bubble to reach the force balance, and the Basset force becomes a lift force in the late rising stage. Furthermore, the Basset force (less than zero) is considered as the resistance in the case of $d_{1}$ = 0.5 mm and $d_{2}$ = 1 mm, where ${\vec{F}}_{Ba}$/(${\vec{F}}_{D}$+${\vec{F}}_{Ba}$) is adopted for the characterization of Basset force contribution. In the case of $d_{3}$ = 2 mm and $d_{4}$ = 4 mm, the direction of Basset force is the same as that of the buoyancy; hence, |${\vec{F}}_{Ba}$/${\vec{F}}_{B}$| is employed for the characterization in such a condition. Figure 10b presents ${\vec{F}}_{Ba}$/(${\vec{F}}_{D}$+${\vec{F}}_{Ba}$) of $d_{1}$ = 0.5 mm (in black) and $d_{2}$ = 1 mm (in red), |${\vec{F}}_{Ba}$/${\vec{F}}_{B}$|of $d_{3}$ = 2 mm (in blue) and $d_{4}$ = 4 mm (in green). The contribution of Basset force is smaller than 0.1 when $d_{b}$ ≥ 1 mm, where the Basset force is considered to be negligible. This is due to the fact that the increase in the bubble size results in a greater buoyancy and acceleration; hence, a larger Basset force occurs at the beginning. Before reaching the force balance, the bubble acceleration reduces to the small scale due to the great Basset force; hence, the contribution of Basset force seems less important in the most later motion.

### 4.4. Two-Phase Density Ratio Effects

**Figure 11:**
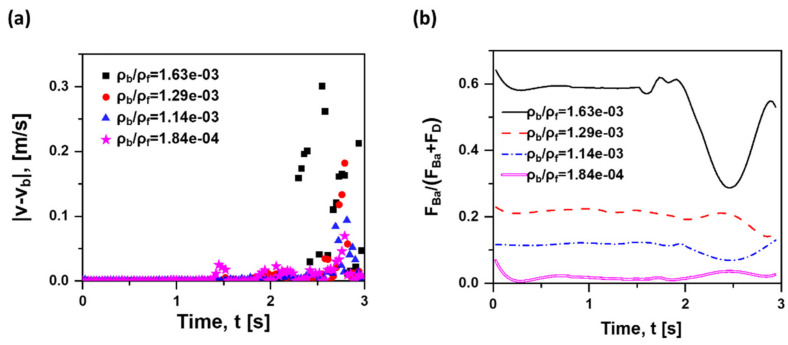
(**a**): Absolute difference in rising velocity between the case with and without Basset force of three density ratios; (**b**)$\;{\vec{F}}_{Ba}$/(${\vec{F}}_{D}$ +${\vec{F}}_{Ba}$ ) over time of three density ratios ($v_{0}$ = 0 m/s, $d_{b}$ = 1 mm, $\rho_{b}$ = 1.29 kg/m^3^ in quiescent water).

The discrete phase remains unchanged, and the density of the fluid varies ($\rho_{f}$ = 7000 kg/m^3^, 1129 kg/m^3^, 1000 kg/m^3^ and 789 kg/m^3^ corresponding to liquid steel at 1873 K, 50% aqueous glycerine solutions at 363 K, water at 293 K and alcohol at 293 K), leading to cases with different density ratios ($\rho_{b}/\rho_{f}$ = 1.84 × 10^−4^, 1.14 × 10^−3^, 1.29 × 10^−3^ and 1.63 × 10^−3^). From Figure 11a, the velocity gap increases with the increase in $\rho_{b}/\rho_{f}$ and time accumulation; that is, there is a more significant effect of Basset force on the cases with a smaller density ratio. This is due to the fact that the decrease in $\rho_{b}/\rho_{f}$ represents a relatively smaller bubble density as to the fluid, where the state of the bubble with smaller inertia can be changed easily, and they are more sensitive to the fluctuating velocity field. According to Stokes law, the suspension velocity where the bubble reached equilibrium is equal to $\frac{2{\pi r}^{2}(\rho_{f}-\rho_{b})g}{9\mu }$. Hence, the larger the density ratio of the bubble in quiescent liquid is, the smaller the suspension velocity. In this instance, the bubble velocity is smaller in the later stage close to equilibrium and the slip velocity becomes smaller; so does the contribution of Basset force on the bubble motion. Furthermore, as the density ratio increases, the time required to reach 95% of the terminal falling velocity (suspension velocity) increases rapidly in the Stokes region (under the creeping flow conditions) [46]. The inertial flow goes the same way [47,48]. As seen in Figure 11b, the contribution of Basset force decreases with the decrease in density ratio. Therefore, a quantitative conclusion can be drawn that the calculation of Basset force can be ignored when the density ratio is smaller than 1.14 × 10^−3^, and its contribution is less than 10%.

### 4.5. Two-Phase Viscosity Ratio Effects

**Figure 12:**
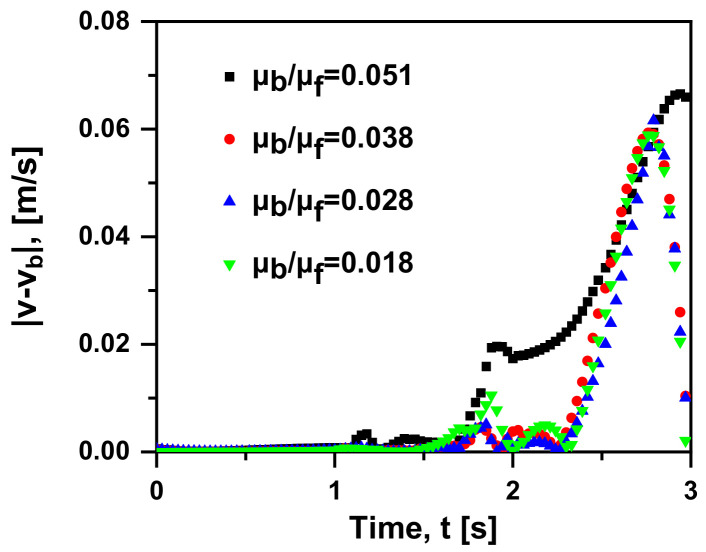
Absolute difference in rising velocity between the case with and without Basset force at four viscosity ratios ($v_{0}$ = 0 m/s, $d_{b}$ = 1 mm, $\rho_{f}$ = 1000 kg/m^3^, $\rho_{b}$ = 1.29 kg/m^3^ in quiescent water).

**Figure 13:**
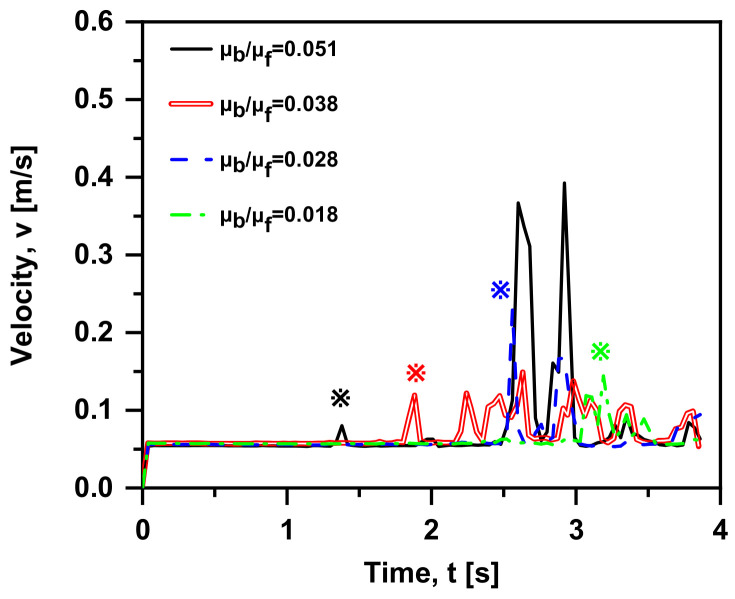
Bubble velocity over time with Basset force at four viscosity ratios ($v_{0}$ = 0 m/s, $d_{b}$ = 1 mm, $\rho_{f}$ = 1000 kg/m^3^, $\rho_{b}$ = 1.29 kg/m^3^ in quiescent water).

**Figure 14:**
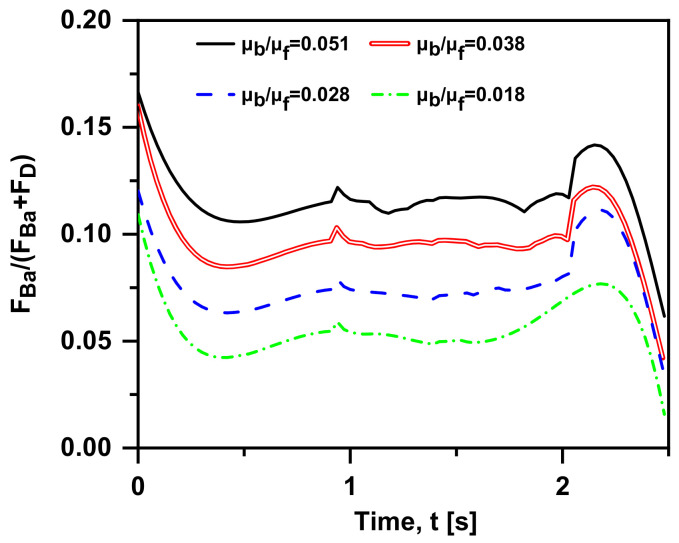
${\vec{F}}_{Ba}$/(${\vec{F}}_{D}$ +${\vec{F}}_{Ba}$ ) over time at four viscosity ratios ($v_{0}$ = 0 m/s, $d_{b}$ = 1 mm, $\rho_{f}$ = 1000 kg/$m^{3}$, $\rho_{b}$ = 1.29 kg/m^3^ in quiescent water).

Admittedly, the physical mechanism of Basset force is an additional unsteady viscous force caused by the lagging development of the boundary layer on the particle surface. In this sub-section, the viscosity of the gas phase remains constant, and different viscosity ratios ($\mu_{b}/\mu_{f}\;$ = 0.051, 0.038, 0.028 and 0.018) are obtained by setting different water viscosities at 293–353 K (3.56 × 10^−4^ Pa·s, 4.69 × 10^−4^ Pa·s, 6.53 × 10^−4^ Pa·s and 1e × 10^−3^ Pa·s). A similar trend can be seen in Figure 12: the velocity gap tends to be zero and then increases to reach the maximum value. Furthermore, in the early motion (t < 2 s), the result with different $\mu_{b}/\mu_{f}$ is less distinctive, that is, the viscosity has little effect on the bubble motion. In order to measure the influence of the force, the bubble velocity changing over time is provided in Figure 13. When t > 1 s, all the cases show the trend of “from increasing to decreasing” repetition. It is appropriate to remark here that velocity peaks (marked as the corresponding star) are reached in all the cases and occur at a later time with the increase in viscosity ratio. In other words, the smaller the viscosity ratio is, the more delayed the effect of Basset force on the bubble motion. Moreover, when the viscosity ratio decreases, the trend of velocity becomes gentler. The primary factor is that the greater viscosity of the fluid leads to the larger shear force per unit velocity, worse fluidity and momentum exchange; hence, the lagging occurs. As shown in Figure 14, when the viscosity ratio is smaller than 0.028, the value of ${\vec{F}}_{Ba}$/(${\vec{F}}_{D}$+${\vec{F}}_{Ba}$) reduces to 0.1, where the Basset force is regarded to be negligible. Furthermore, the drag coefficient on a Newtonian fluid sphere translating with a constant velocity in another immiscible Newtonian fluid is given theoretically [15]:

(14) $$C_{D}=\left(\frac{24}{Re}\right)Y$$

where $Y=\frac{2+3\frac{\mu_{b}}{\mu_{f}}}{3+3\frac{\mu_{b}}{\mu_{f}}}$ and $Re=\frac{\rho_{f}vd_{b}}{\mu_{f}}$. In terms of a gas bubble, the viscosity ratio tends to be zero and *Y* is equal to 2/3 under this instance. Therefore, in the case of identical $\mu_{b}$, the drag coefficient decreases with the increase in the viscosity ratio, leading to a smaller drag force and a greater contribution of Basset force. However, the range of this model is narrow due to neglecting bubble shape in our work. The bubble is usually subjected to buoyancy, gravity, viscous force, virtual mass force and Basset force, and the shape of the bubble depends on the balance of these forces. A viscous shear effect is caused by the viscosity difference at the bubble–fluids interphase, which overcomes the resistance induced by surface tension to form bubble deformation. The greater the viscosity ratio of the gas phase to the liquid phase is, the more severe the bubble deformation.

## 5. Conclusions

Due to its major importance to both fundamental research and industrial application, the description of bubble motion under complex flow conditions is becoming more and more accurate, and the research on the force analysis has become of more concern to scholars. Notwithstanding the fact that it is not at all justifiable to ignore the Basset force term in the calculation, considering the complex origin and calculation of Basset force itself, this practice is commonly found in many studies. Furthermore, a complete solution involving all potential influence factors is not yet available. Therefore, for the first time, a qualitative and quantitative research on the effect of Basset force on single bubble motion is conducted by a coupled CFD-DEM approach. Overall, the conclusions are as follows:

(a) Basset force represents the influence of unsteady viscosity in the process of motion; hence, it should not be ignored. In particular, by the method of Fourier series representation, Basset force on the particles/bubbles is very small and can be ignored for the high-frequency component in the turbulent flow field.
(b) In order to realize the unsteady coupling process of the bubble, a coupled CFD-DEM model with Basset force is constructed, and there is good agreement with the experimental and numerical results.
(c) Five potential influence factors are studied in this work: initial velocity, releasing depth, bubble size, density ratio and viscosity ratio. In our cases, ${\vec{F}}_{Ba}$/(${\vec{F}}_{D}$+${\vec{F}}_{Ba}$) and |${\vec{F}}_{Ba}$/${\vec{F}}_{B}$| are employed to quantify the contribution of Basset force, and the results indicated that:
  (1) Basset force is a matter of less significance to the bubble with higher initial velocity due to a larger order of magnitude of drag force than Basset force, and the effect of Basset force can be ignored in the case of $v_{0}$ ≥ 20 m/s.
  (2) It is evident that the shallower the releasing depth is, the smaller the pressure at the initial position is, resulting in the lower impact of Basset force on the bubble motion. Or more quantitatively, when the releasing depth is smaller than 6.9 m, its effect can be negligible.
  (3) As for the bubble size, the effect of Basset force on the bubble motion decreases with the diameter increasing, and the Basset force is considered to be overlooked when the bubble diameter is larger than 1 mm.
  (4) When the density ratio becomes smaller, there is a more intensified effect of Basset force on the bubble motion due to the low inertia of the bubble. Moreover, the calculation of Basset force can be ignored when the density ratio is greater than 1.14 × 10^−3^.
  (5) In terms of the smaller viscosity ratio, the effect of Basset force on the bubble motion becomes more delayed, because the greater fluid viscosity leads to a larger shear force per unit velocity and worse momentum exchange. When the viscosity ratio is smaller than 0.028, the contribution of Basset force reduces to 0.1, and it is regarded to be negligible.

## Figures and Tables

**Table 1:** Different conditions considering the value of n.

n	${\vec{F}}_{Ba}=BC_{B}\int_{t_{0}}^{t}\frac{b{t^{\prime }}^{n}}{\sqrt{t-t^{\prime }}}dt^{\prime }$	Explanation	
0	$2\mathrm{bB}C_{B}t^{1/2}$	The particle moves at constant acceleration relative to the fluid. Basset force increases monotonically with t and can reach a considerable order of magnitude given enough large t. (When b = 0, the particle will move at a uniform speed relative to the fluid and ${\vec{F}}_{Ba}=$0)	
1	$\frac{4}{3}$ $\mathrm{bB}C_{B}t^{3/2}$	The particle makes an aperiodic variable acceleration motion relative to the fluid.	${\vec{F}}_{Ba}$ varies with t according to the law of 3/2 power.
k	$\frac{2^{k+1}\cdot k!}{1\cdot 3\cdot 5\cdot \cdot \cdot \left(2k+1\right)}$ $\mathrm{bB}C_{B}t^{\left(k+1\right)/2}$		$F_{Ba}$ varies with t according to the law of 1/2 power increase.

**Table 2:** Physical properties in the experiment.

Material	Density (kg/m^3^)	Viscosity (Pa·s)
Air	1.29	1.8 × 10^−5^
Water	1000	1.0 × 10^−3^

**Table 3:** Cases of mesh independence.

Mesh	Nodes	Hexahedral Cells	GCI (%)
1.1 mm grid size (mesh 1)	10,528,218	10,417,488	0.52
1.2 mm grid size (mesh 2)	8,194,765	8,101,296	1.71
1.5 mm grid size (mesh 3)	4,272,020	4,211,392	2.81
2.0 mm grid size (mesh 4)	1,859,688	1,824,977	5.06
3.0 mm grid size (mesh 5)	570,741	554,944	10.05

## Data Availability

The data that support the findings of this study are available from the corresponding author upon reasonable request.

## Supplementary Materials

The following supporting information can be downloaded at: https://www.mdpi.com/article/10.3390/ma15155461/s1, Table S1. Bubble-fluid interphase forces in coupling interface.

## Nomenclature

$C_{B}$	correction coefficient
$C_{D}$	drag coefficient
d	diameter, m
${\vec{F}}_{Ba}$	Basset force, N
${\vec{F}}_{D}$	drag force, N
${\vec{F}}_{c,ij}$	contact force, N
${\vec{F}}_{nc,ik}$	noncontact force, N
${\vec{F}}_{bf}$	Bubble–fluid interaction force, N
${\vec{F}}_{G}$	gravity force, N
${\vec{f}}_{\mathrm{bf}}$	reacting force of bubble–fluid interactions, N
h	releasing depth, m
I	moment of inertia
$\overset{=}{I}$	identity matrix
${\vec{M}}_{r,ij}$	rolling friction moments, N·m
${\vec{M}}_{t,ij}$	tangential friction moments, N·m
m	mass, kg
P	pressure, Pa
Q	flow rate, L/h
Re	Reynolds number
t	time, s
$t_{0}$	initial time, s
$t_{s}$	time to reach liquid surface, s
$t_{i}-t_{e}$	the time differences between the simulation and the experiment, s
$\vec{u}$	fluid velocity, m/s
V	volume, m^3^
$\vec{v}$	bubble/particle velocity, m/s
$v_{b}$	bubble velocity in the case with Basset force, m/s
$\vec{v_{i}}$	translation velocity of the bubble, m/s
$v_{r}$	rising velocity, m/s
$v_{i}-v_{e}$	the velocity differences between the simulation and the experiment, m/s
*Greek symbols*	
a	volume fraction,
ρ	density, kg/m^3^
μ	dynamic viscosity, Pa·s
${\vec{\omega }}_{i}$	angular velocity of the bubble, rad/s
$\mu_{q}$	shear viscosity of phase-q, Pa·s
$\lambda_{q}$	bulk viscosity of phase-q, Pa·s
*Subscripts*	
b	bubble phase
f	fluid phase
q	phase-q
