# Supplementary material for: A Coupled CFD-DEM Study on the Effect of Basset Force Aimed at the Motion of a Single Bubble
Source: Materials (Basel). 2022 Aug 8;15(15):5461. doi: 10.3390/ma15155461 (PMC9369531; doi:10.3390/ma15155461)
Supplement: Supplementary file 1 [file materials-15-05461-s001.zip › materials-1829990-supplementary.pdf]

Supplementary

The models of interaction forces are shown in the **Table S1**.

Table S1. Bubble-fluid interphase forces in coupling interface.

Models	Correlations
Drag	$\vec{F}_D = C_D \frac{1}{8} \rho_f \pi d_b^2 (\vec{u} - \vec{v}) \lvert \vec{u} - \vec{v} \rvert$
Pressure gradient force	$\vec{F}_p = -V_b \nabla p$
Viscous stress force	$\vec{F}_{\nabla \cdot \tau, i} = -V_b \nabla \cdot \tau$
Reynolds stress force	$\vec{F}_{\nabla \cdot (\rho \overline{u_i' u_j'}), i} = -V_b \nabla \cdot (\rho \overline{u_i' u_j'})$
Capillary force	$\vec{F}_{s,i} = V_b \vec{f_s} = V_b \sigma \cdot \kappa \cdot \nabla \phi$
Saffman lift force	$\vec{F}_{saffman} = \mathrm{m_b} \frac{2 \mathrm{K} \rho_f d_{ij} \nu^{1/2}}{\rho_b d_b (d_{lk} d_{kl})^{1/4}} (\vec{u} - \vec{v}), \ \mathrm{K} = 2.594$
Magnus lift force	$\vec{F}_{magnus} = 0.5 A_b C_{RL} \rho_f \frac{\lvert \vec{u} - \vec{v} \rvert}{\lvert \vec{\Omega} \rvert} (\vec{u} - \vec{v}) \times \vec{\Omega}$
Basset force	$\vec{F}_{Ba} = \frac{3}{2} d_b^2 \sqrt{\pi \rho_f \mu_f} \int_{t_0}^{t} \frac{\frac{d(\vec{u} - \vec{v})}{dt'}}{\sqrt{t - t'}} dt'$

The detailed codes compiled into the coupling interface is as follows.

```
void compute_particle_forces_step_by_step(Tracked_Particle *tp, Injection *I, cxboolean
fluid_source_terms)
{
```

```
#if !RP_HOST
  Particle *pp;
  real buoyancyForce[ND_ND];
  real dragForce[ND_ND];
  real bassetForce[ND_ND];
  real t = CURRENT_TIME;
  real dt = CURRENT_TIMESTEP;
  real relVel[ND_ND], relVelMag, rv[ND_ND], relVel1[ND_ND];
  Thread *ct, *vt;
  cell_t c;
  real factor;
  real Re;
  real Cd;

  loop(pp, I->p)
  {
      c = P_CELL(pp);
      vt = ct = P_CELL_THREAD(pp);
      /* for euler/euler && DDPM we need the thread holding the velocity */
      if (mp_mfluid)
          vt = DPM_THREAD(ct, NULL);

#if (RampantReleaseMajor <= 17)
      init_tracked_particle(tp, pp, dpm_par.unsteady_tracking, FALSE, FALSE);
#endif

#if (RampantReleaseMajor == 18)
# if (RampantReleaseMinor >= 1)
      init_tracked_particle(tp, pp, dpm_par.unsteady_tracking, FALSE, TRUE);
# else
      init_tracked_particle(tp, pp, dpm_par.unsteady_tracking, FALSE, FALSE, TRUE);
# endif
#endif

#if (RampantReleaseMajor >= 19)
      init_tracked_particle(tp, pp, dpm_par.unsteady_tracking, FALSE, TRUE);
#endif



      /* Buoyancy force */
      if (M_gravity_p)
      {
          factor = -P_N(pp) * C_R(c, vt) * DPM_VOLUME(P_DIAM(pp)); /* Volume has been
stored via diameter assuming spherical particles */
```

```
        NV_VS(buoyancyForce, = , M_gravity, *, factor);
    }
    else
        NV_S(buoyancyForce, = , 0.0);

    ND_V(P_DEM_FORCE_X(pp), P_DEM_FORCE_Y(pp), P_DEM_FORCE_Z(pp), = , buoyancyForce);

    /* Drag Force */

    NV_D(relVel, = , C_U(c, vt), C_V(c, vt), C_W(c, vt));
    NV_V(relVel, -= , P_VEL(pp));

    relVelMag = NV_MAG(relVel);

    Re = (C_R(c, vt) * relVelMag * P_DIAM(pp)) / C_MU_L(c, vt);

    if (Re <= 0.55)
        Cd = 24.0 / Re;
    else if (Re <= 987.0)
        Cd = 24.0 * (1.0 + 0.15 * pow(Re, 0.687)) / Re;
    else
        Cd = 0.44;

    factor = P_N(pp) * (0.5 * Cd * C_R(c, vt) * relVelMag * P_PROJECTED_AREA(pp));

    NV_VS(dragForce, = , relVel, *, factor);

    ND_V(P_DEM_FORCE_X(pp), P_DEM_FORCE_Y(pp), P_DEM_FORCE_Z(pp), += , dragForce);

    /*Basset force*/

    /* acceleration of fluid */
    NV_D(relVel1, = , C_U(c, vt), C_V(c, vt), C_W(c, vt));
    NV_V(relVel1, -= , P_VEL(pp));

    NV_VS(rv, = , relVel1, /, dt);

    if (P_RHO(pp) < 1000.0 && M_gravity_p)
    {
        factor = P_N(pp)* 3.0 * pow(P_DIAM(pp), 2.0) * sqrt(M_PI * C_R(c, vt) *
C_MU_L(c, vt) * t);
        NV_VS(bassetForce, =, rv, *, factor);
    }
```

```
        else
            NV_S(bassetForce, = , 0.0);

        ND_V(P_DEM_FORCE_X(pp), P_DEM_FORCE_Y(pp), P_DEM_FORCE_Z(pp), += , bassetForce);

        /* if (fluid_source_terms) */
        /* not yet implemented */

        /* Ignoring torque for the time being */
        /* Scaling by P_N may need to be SQR(P_N) */
        ND_S(P_DEM_TORQUE_X(pp),P_DEM_TORQUE_Y(pp),P_DEM_TORQUE_Z(pp),=,0.0);
      }
#endif /* !RP_HOST */
}


void compute_forces_on_particles(EDEM_Coupling edem_coupling, cxboolean
fluid_source_terms)
{
#if !RP_HOST
    int i_pro;
    Injection *Ip;
#endif

    if (edem_coupling.num_particle_prototypes <= 0)
    {
        Message0("\nWARNING: Particle data has not been read from EDEM yet.\n\n");
        return;
    }

    if (NULLP(edem_coupling.injections) || NULLP(edem_coupling.injection_names))
    {
        Message0("\nWARNING: Injections for EDEM particles have not been set
up.\n\n");
        return;
    }

#if !RP_HOST

    for (i_pro = 0; i_pro < edem_coupling.num_particle_prototypes; i_pro++)
    {
        Ip = edem_coupling.injections[i_pro];

        if (NNULLP(Ip->p))
```

```
        {
            Tracked_Particle tp_init = { 0 };
            Tracked_Particle *tp = &tp_init;

            alloc_tracked_particle_memory(tp);
            alloc_tp_pvars(tp, Ip);

            if (DEM_DRAG_FLUENT_P(edem_coupling) ||
DEM_HEAT_TRANSFER_FLUENT_P(edem_coupling))
                compute_particle_forces_using_Fluent(tp, Ip, fluid_source_terms);

            if (!DEM_DRAG_FLUENT_P(edem_coupling))
                compute_particle_forces_step_by_step(tp, Ip, fluid_source_terms);

            free_tp_pvars(tp);
            free_tracked_particle_memory(tp);
        }
    }
#endif /* !RP_HOST */
}
```
